# S100B immunization triggers NFκB and complement activation in an autoimmune glaucoma model

**DOI:** 10.1038/s41598-018-28183-6

**Published:** 2018-06-29

**Authors:** Sabrina Reinehr, Jacqueline Reinhard, Marcel Gandej, Ivo Gottschalk, Gesa Stute, Andreas Faissner, H. Burkhard Dick, Stephanie C. Joachim

**Affiliations:** 1 Experimental Eye Research Institute, University Eye Hospital, Ruhr-University Bochum, In der Schornau 23-25, 44892 Bochum, Germany; 2 Department of Cell Morphology and Molecular Neurobiology, Faculty of Biology and Biotechnology, Ruhr-University Bochum, Universitaetsstrasse 150, 44780 Bochum, Germany

## Abstract

In glaucoma, latest studies revealed an involvement of the complement system with and without an elevated intraocular pressure. In the experimental autoimmune glaucoma model, immunization with antigens, such as S100B, lead to retinal ganglion cell (RGC) loss and optic nerve degeneration after 28 days. Here, we investigated the timeline of progression of the complement system, toll-like-receptor 4 (TLR4), and the transcription factor nucleus factor-kappa B (NFκB). Therefore, rats were immunized with S100B protein (S100) and analyzed at 3, 7, and 14 days. RGC numbers were comparable at all points in time, whereas a destruction of S100 optic nerves was noted at 14 days. A significant increase of mannose binding lectin (MBL) was observed in S100 retinas at 3 days. Subsequently, significantly more MBL^+^ cells were seen in S100 optic nerves at 7 and 14 days. Accordingly, C3 was upregulated in S100 retinas at 14 days. An increase of interleukin-1 beta was noted in S100 aqueous humor samples at 7 days. In this study, activation of complement system via the lectin pathway was obvious. However, no TLR4 alterations were noted in S100 retinas and optic nerves. Interestingly, a significant NFκB increase was observed in S100 retinas at 7 and 14 days. We assume that NFκB activation might be triggered via MBL leading to glaucomatous damage.

## Introduction

Glaucoma is defined as a progressive optic neuropathy with changes at the optic nerve head, gradual retinal ganglion cell (RGC) death, and visual field loss^1^. An elevated intraocular pressure (IOP) is still considered the main risk factor^1^, but there are increasing evidences that other pathological factors are involved. Some glaucoma patients, who do not have an elevated IOP, also show glaucomatous damage^2^. Glaucoma is a multifactorial disease and besides other factors recent studies revealed a contribution of immunological processes^3–5^. To analyze these mechanisms, the experimental autoimmune glaucoma model (EAG) was developed. Here, immunization with ocular antigens leads to RGC loss and optic nerve degeneration without IOP elevation^6–9^. In this model, autoreactive antibodies were detected in the retinas as well as in optic nerves^6,10^. Furthermore, an increase in microglia cell numbers, the macrophages of the central nervous system (CNS), was noted in these retinas and optic nerves^8,11^. This raises the question whether the microglia are an epiphenomenon or part of the degeneration process. For instance, retinal microglia can produce complement system proteins^12–14^. The complement system, as part of the innate immune system, is activated via three distinct routes. The classical pathway can be initiated through the protein C1q, while the mannose-binding-lectin (MBL) induces the lectin pathway. The alternative pathway is spontaneously activated through cleavage of C3b. All three ways are assembled in the terminal pathway, which starts with the protein C3. Finally, the membrane attack complex (MAC) forms a pore in the target cell and forces its lysis. In the last years, studies showed a contribution of the complement system in glaucoma disease, e.g. depositions of complement components were observed in the human glaucomatous retina^15,16^. Those depositions were also noted in ocular hypertension (OHT) animal models^17,18^. Our group could also show an IOP-independent activation of the complement system in retinas and optic nerves^19^.

One regulator of the innate immune system is the transcription factor nucleus factor-kappa-light-chain-enhancer of activated B cells (NFκB). It controls several cellular mechanisms such as proliferation, differentiation, survival, and apoptosis^20^. In unstimulated cells, NFκB accumulates in the cytoplasm. After stimulation, the inhibitory protein IκBα dissociates from the NFκB complex and the transcription factor translocates into the nucleus to initiate the expression of various target genes^21,22^. Therefore, we analyze if NFκB activation can be initialized via toll-like-receptors (TLRs). This receptor family is located on microglia, dendritic cells, and macrophages^23,24^. TLR4 is known to play a role in neuronal cell death in the CNS^25^. Also, in glaucoma, an increased TLR4 expression seems to be involved in neurodegenerative processes. In human glaucoma donor eyes as well as in OHT animal models, an increase of TLRs was noted^26^. In optic nerve injury models, an activation of the TLR4/NFκB pathway was also observed^27,28^.

We propose an activation of the complement system as well as an enhanced TLR4/NFκB pathway signaling in retinas and optic nerves of the EAG model before cell loss. To investigate this hypothesis, several cell types were evaluated via immunohistology and quantitative real-time PCR (qRT-PCR) 3, 7, and 14 days after immunization. Additionally, the levels of interleukin-1 beta (IL-1β) were measured in serum and aqueous humor samples at all points in time.

We did not find alterations in the number of RGCs, while an optic nerve degeneration was seen after 14 days in the S100 group. Furthermore, we detected an activation of the complement system through the lectin pathway after 3 days, while the expression of TLR4 was not affected at all investigated points in time. However, we noted an increased number of NFκB^+^ cells in the S100 retinas. Additionally, an increase of IL-1β was observed in S100 aqueous humor samples at 7 days.

## Results

### No apoptotic retinal ganglion cells, but optic nerve degeneration

**Figure 1:**
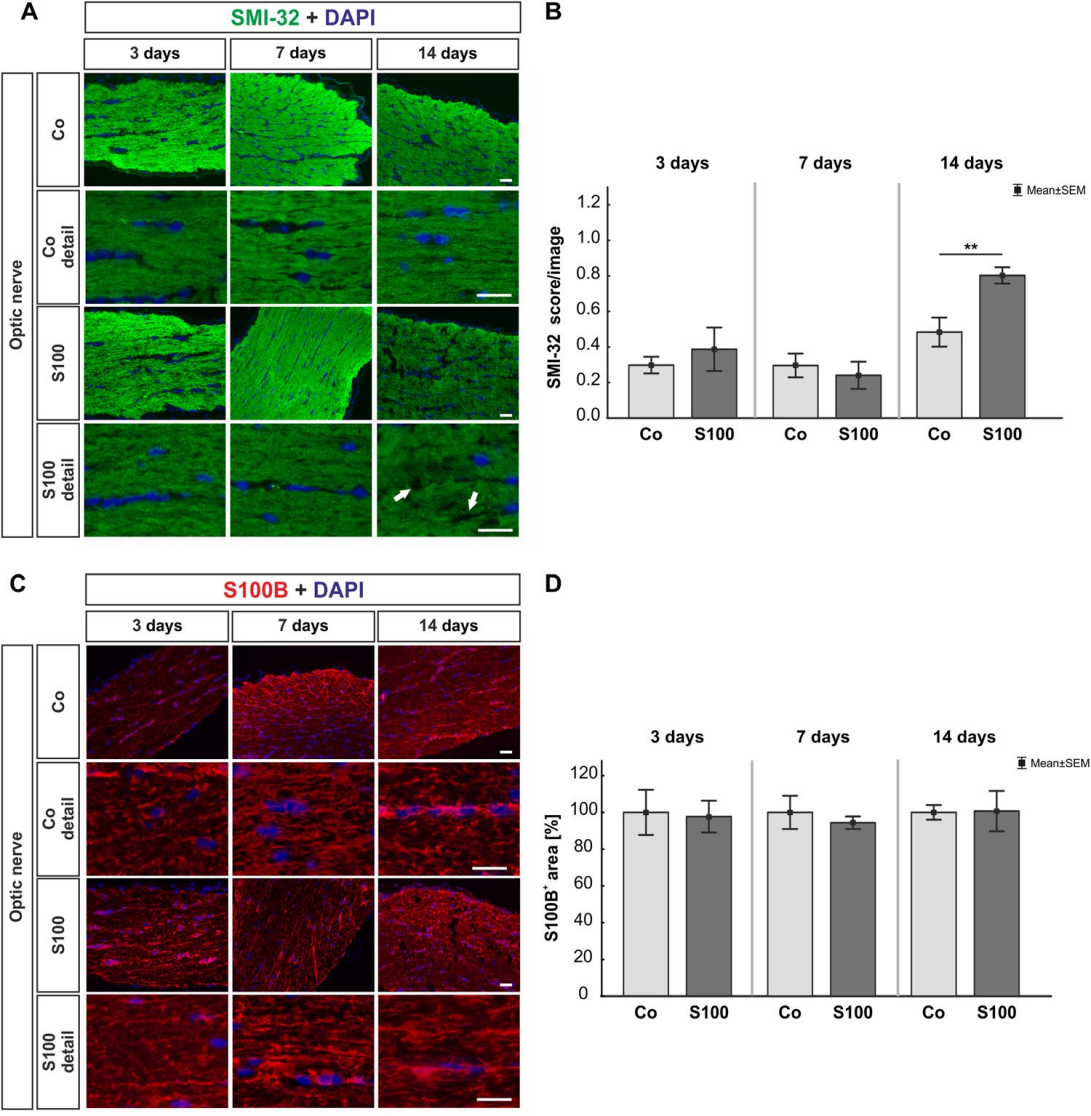
Optic nerve degeneration. **(A)** At 3, 7, and 14 days, neurofilaments were labeled with anti-SMI-32 (green). Cell nuclei were stained with DAPI (blue). Arrows point towards disrupted filaments. **(B)** No changes were noted in the SMI-32 score 3 and 7 days after immunization (p > 0.05). However, a significant increase of the SMI-32 score was observed in S100 optic nerves at 14 days (p = 0.007). **(C)** Astrocytes in the optic nerves were labeled with an anti-S100B antibody (red) and DAPI (blue). **(D)** We did not note any changes regarding the S100B^+^ area in the S100 optic nerves compared to controls (p > 0.05). Values are mean ± SEM. Scale bars: 20 µm.

At 14 days, Brn-3a stained retinal whole mounts revealed no statistically significant alterations in the S100 group (p > 0.05, supplement Fig. 1A,B). However, in total as well as in the three separate regions of the whole mounts (central, middle, peripheral), a slight decline of about 10% of RGCs was observable. The number of RGCs on retinal cross-sections was comparable in S100 and Co animals at 3, 7, and 14 days (p > 0.05, supplement Fig. 1C,D). Also, no changes were observed in cleaved caspase^+^ RGCs in the S100 group after all points in time (p > 0.05, supplement Fig. 1C,E). QRT-PCR revealed no alterations in the expression level of *Pou4f1* and *Bax/Bcl-2* (p > 0.05, supplement Fig. 1F,G, Table 1). In regard to SMI-32 labeling in the optic nerve, no changes occurred in the S100 group after 3 and 7 days (p > 0.05, Fig. 1A,B). However, a significantly higher SMI-32 score was noted in S100 animals at 14 days (p = 0.007). The S100B optic nerve staining showed no alterations between both groups throughout the study (p > 0.05, Fig. 1C,D).

**Table 1:** Analyses of mRNA levels via quantitative real-time PCR. Significant values are marked in bold.

	3 days	7 days	14 days
*Bax/Bcl-2*	0.99 (0.67–1.44)	0.89 (0.76–1.17)	0.84 (0.77–0.96)
P-value	0.9	0.4	0.07
*C3*	0.57 (0.20–1.16)	0.73 (0.53–1.07)	1.56 (1.33–1.87)
P-value	0.1	0.2	**<0.001**
*C5*	0.84 (0.50–1.31)	1.47 (1.06–1.98)	0.79 (0.62–0.96)
P-value	0.5	0.3	0.3
*Factor B*	2.22 (1.41–3.82)	0.76 (0.54–1.26)	0.54 (0.37–1.09)
P-value	**0.02**	0.3	0.3
*Mbl*	1.66 (1.31–2.22)	0.91 (0.73–1.09)	0.48 (0.32–0.839)
P-value	**0.047**	0.6	0.08
*NEMO*	0.76 (0.55–1.05)	1.00 (0.87–1.21)	0.93 (0.86–0.99)
P-value	0.09	0.99	0.2
*Nfκb*	0.83 (0.57–1.31)	1.33 (1.06–1.67)	0.97 (0.86–1.07)
P-value	0.4	0.2	0.8
*Myd88*	1.48 (0.99–2.44)	0.86 (0.72–1.13)	0.92 (0.75–1.29)
P-value	0.1	0.3	0.7
*Pou4f1*	0.80 (0.52–1.22)	1.16 (0.90–1.42)	0.94 (0.85–1.08)
P-value	0.3	0.3	0.4
*Tlr4*	1.04 (0.72–1.50)	1.45 (0.89–2.13)	1.11 (0.89–1.35)
P-value	0.8	0.4	0.5

### Slight complement activation via the lectin pathway in retinas after immunization

**Figure 2:**
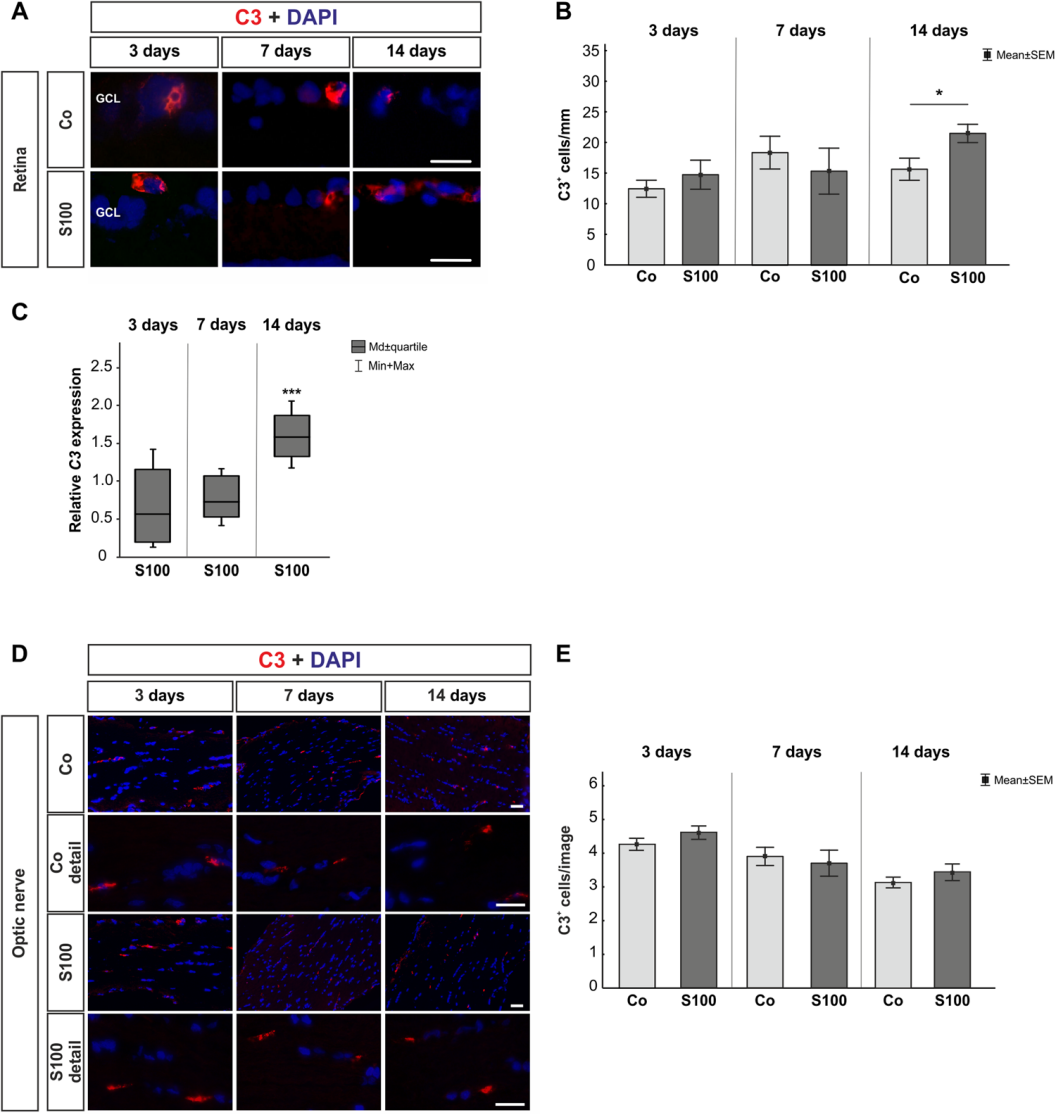
Mild C3 activation. **(A)** To evaluate an activation of C3, retinal cross-sections were stained with anti-C3 (red) and DAPI (cell nuclei, blue) 3, 7, and 14 after immunization. **(B)** No alterations in C3^+^ cell numbers were noted in the S100 group after 3 and 7 days (p > 0.05). After 14 days, significantly more C3 depositions were observed (p = 0.04). **(C)** The *C3* mRNA expression level showed no alterations 3 and 7 days after immunization (p > 0.05). A significant higher level of *C3* mRNA was observed at 14 days (p < 0.001). **(D)** Anti-C3 staining (red) of the optic nerves was performed 3, 7, and 14 days after immunization. Cell nuclei were labeled with DAPI (blue). **(E)** No changes were revealed at all points in time (p > 0.05). Abbreviation: GCL = ganglion cell layer. Values are mean ± SEM for immunohistology and median ± quartile + maximum/minimum for qRT-PCR. Scale bars: 20 µm.

For retinal C3, no changes were noted in the S100 group after 3 and 7 days (p > 0.05, Fig. 2A,B). Also, no alterations were seen in *C3* mRNA expression levels at these points in time (p > 0.05, Fig. 2C). At 14 days, significantly more C3^+^ cells could be observed in the S100 animals (p = 0.04). Additionally, a significant upregulation of *C3* mRNA was detected at this point in time (p < 0.001, Table 1). C3 cell numbers on optic nerves were comparable in both groups throughout the study (p > 0.05, Fig. 2D,E).

In regard to retinal MAC expression, the immunohistological data revealed no alterations in the S100 group at all points in time (p > 0.05, supplement Fig. 2A,B). MAC was often co-localized with Brn-3a^+^ cells (supplement Fig. 3). Also, the *C5* mRNA expression remained unaltered at 3, 7, and 14 days (p > 0.05, supplement Fig. 2C). In the optic nerves, MAC staining was comparable in both groups at all points in time (p > 0.05, supplement Fig. 2D,E).

**Figure 3:**
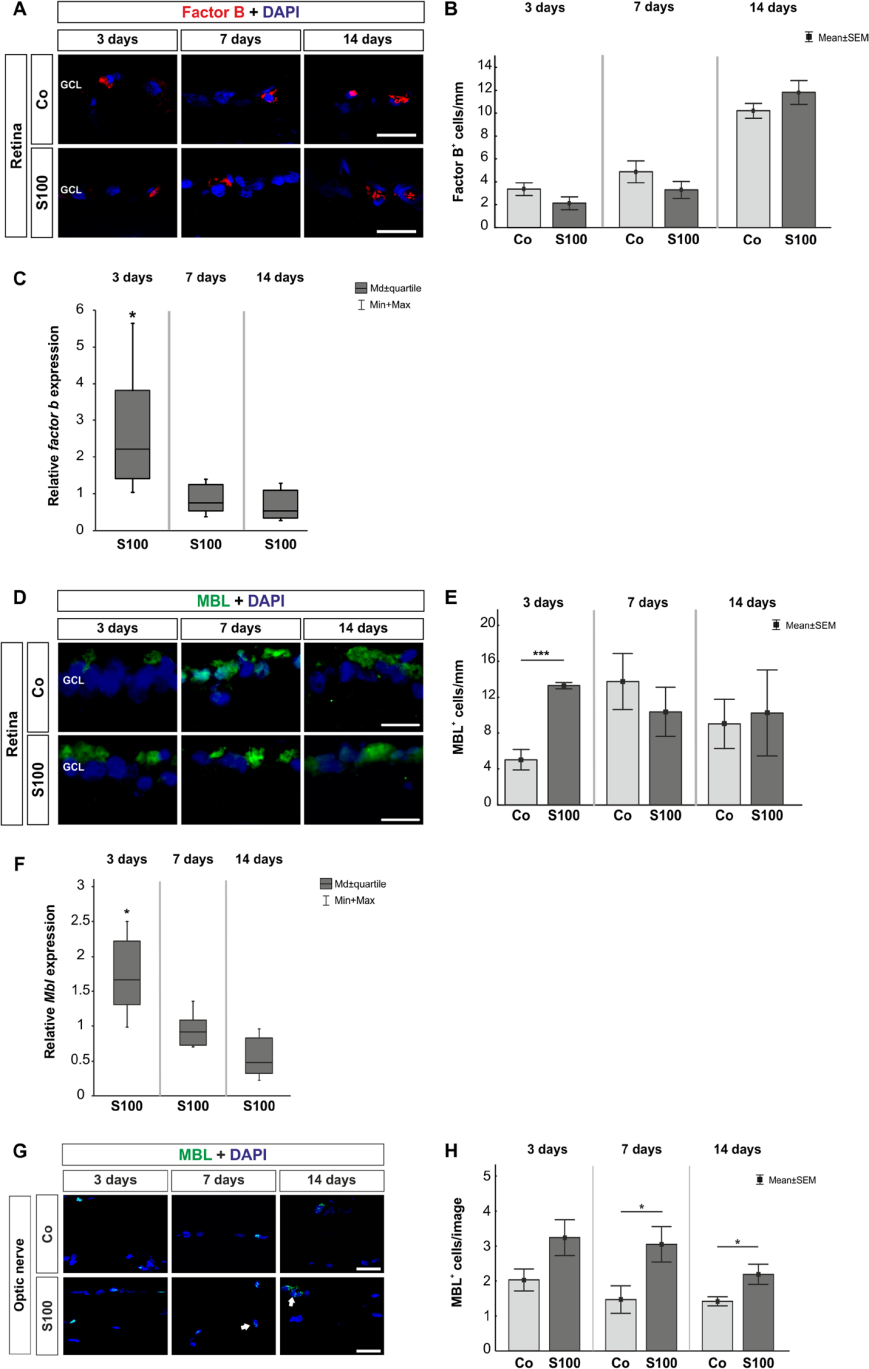
Activation through the lectin pathway. **(A)** Retinas were labeled with an antibody against factor B (red) 3, 7, and 14 days after immunization. Cell nuclei were stained with DAPI (blue). **(B)** No alterations in factor B^+^ cell numbers were noted in S100 animals compared to controls at all points in time (p > 0.05). **(C)** The qRT-PCR analyses revealed an upregulation of *factor b* mRNA at 3 days (p = 0.02). No changes were observed at 7 and 14 days (p > 0.05). **(D)** Retinal cross-sections were stained with anti-MBL (green) and DAPI (blue) at 3, 7, and 14 days. **(E)** At 3 days, significantly more MBL^+^ cells were observed in S100 retinas (p < 0.0001). No changes were noted later on (p > 0.05). **(F)** Expression levels of *Mbl* measured with qRT-PCR at 3, 7, and 14 days. At 3 days, a significant increase of *Mbl* mRNA expression was detected in S100 retinas (p = 0.048). *Mbl* went back to control level later on (p > 0.05). **(G)** Optic nerve sections were labeled with an anti-MBL antibody (green) and cell nuclei with DAPI (blue) 3, 7, and 14 days after immunization. **(H)** The number of MBL^+^ cells remained unaltered in S100 optic nerves at 3 days (p > 0.05). After 7 days, significantly more MBL^+^ cells were noted in the S100 group (p = 0.03) and were still present at 14 days (p = 0.02). Abbreviation: GCL = ganglion cell layer. Values are mean ± SEM for immunohistology and median ± quartile + maximum/minimum for qRT-PCR. Scale bars: 20 µm.

Regarding retinal factor B, no alterations were observed in the S100 animals at 3, 7, and 14 days (p > 0.05, Fig. 3A,B). Interestingly, an upregulation of *factor b* mRNA was noted in the S100 retinas at 3 days (p = 0.02, Fig. 3C, Table 1). At 7 and 14 days, the expression went back to control level (p > 0.05).

Significantly more MBL^+^ cells were noted in the S100 retinas after 3 days (p < 0.0001, Fig. 3D,E). MBL was often co-localized with active microglia (supplement Fig. 3). Additionally, the qRT-PCR analysis revealed an upregulation of *Mbl* mRNA at this point in time (p = 0.047, Fig. 3F). At 7 days, the number of MBL^+^ cells in both groups was similar (p > 0.05). This was still the case at 14 days (p > 0.05). No changes were observed regarding *Mbl* expression at 7 and 14 days (p > 0.05, Table 1). In the optic nerves, the number of MBL^+^ cells remained unaltered after 3 days (p > 0.05, Fig. 3G,H). At 7 days, significantly more MBL^+^ cells were observed in the S100 optic nerves (p = 0.03). An increased number of MBL^+^ cells was still noted after 14 days (p = 0.02).

### Increase of retinal NFκB

**Figure 4:**
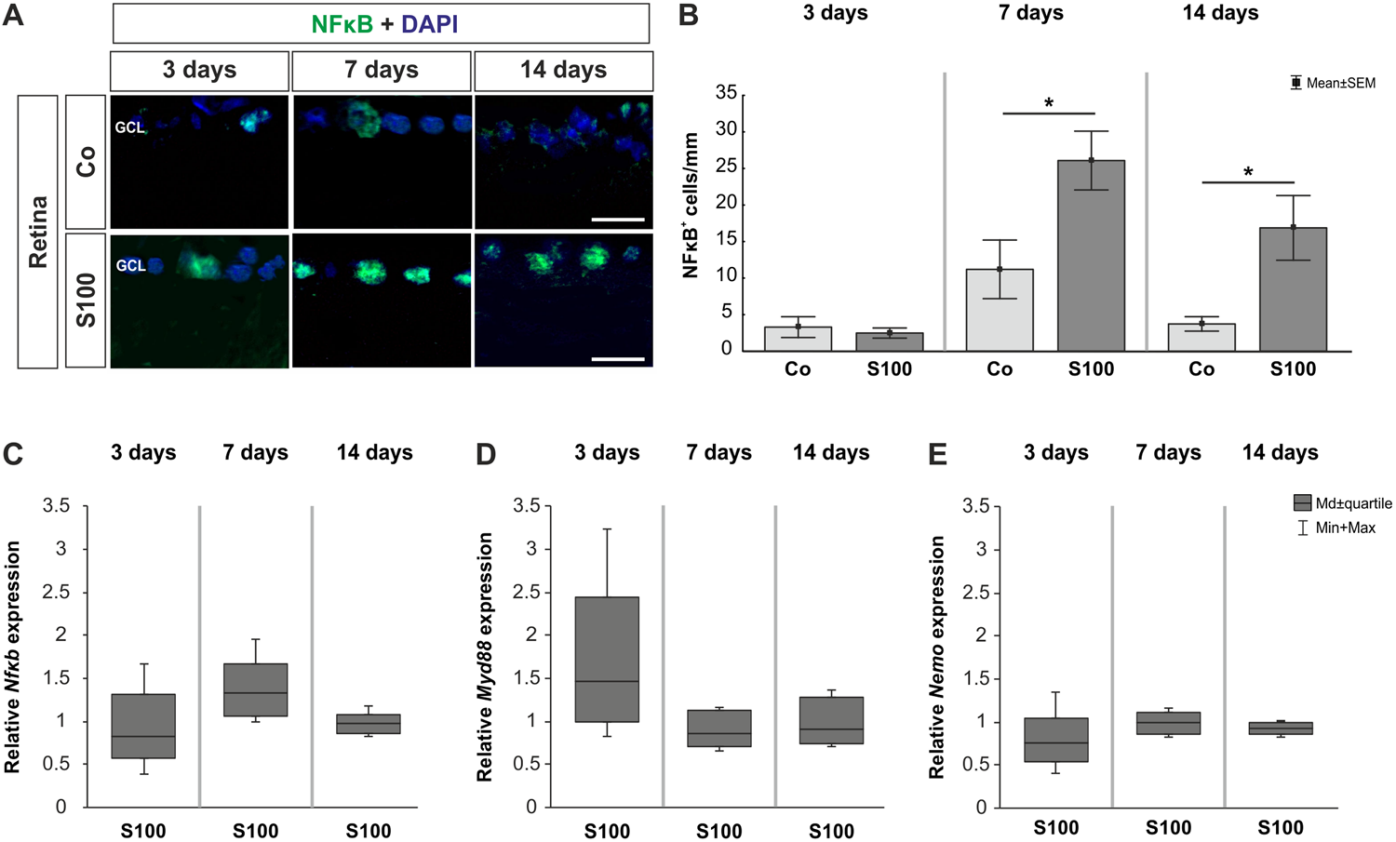
Increased NFκB staining. **(A)** Retinas were labeled with an anti-NFκB antibody (green) and DAPI (blue). **(B)** At 3 days, no changes were observed in regard of the number of NFκB^+^ cells in the S100 group (p > 0.05). After 7 days, significantly more NFκB^+^ cells were noted in S100 animals (p = 0.03). A significant increase of NFκB^+^ cells was still revealed in this group after 14 days (p = 0.03). **(C)** QRT-PCR analysis revealed no differences in *Nfκb* mRNA expression at all points in time (p > 0.05). However, a slight trend towards an upregulation could be observed at 7 days (p = 0.18). **(D)** The expression levels of *Myd88* showed a trend towards an upregulation at 3 days (p = 0.1). At 7 and 14 days, no alterations were noted (p > 0.05). **(E)** At all points in time, the expression of *NEMO* mRNA remained unchanged (p > 0.05). Abbreviation: GCL = ganglion cell layer. Values are mean ± SEM for immunohistology and median ± quartile + maximum/minimum for qRT-PCR. Scale bars: 20 µm.

At 3 days, no changes were noted in regard to NFκB labeling in the S100 group (p > 0.05). More NFκB^+^ cells were observed after 7 days (p = 0.03). Also, a higher number of NFκB^+^ cells was revealed in the S100 retinas at 14 days (p = 0.03, Fig. 4A,B). The qRT-PCR data showed no alterations in *Nfκb* expression after 3 and 14 days (p > 0.05, Table 1), while a slight trend pointed towards an upregulation was noted at 7 days (p = 0.18, Fig. 4C). Furthermore, analyses of *Myd88* expression levels revealed a slight upregulation at 3 days (p = 0.1, Table 1). At 7 and 14 days, the expression of *Myd88* remained unaltered (p > 0.05, Fig. 4D). The mRNA expression levels of *NEMO* showed no changes at all points in time (p > 0.05, Fig. 4E, Table 1).

### No TLR4 alterations in retinas and optic nerves

**Figure 5:**
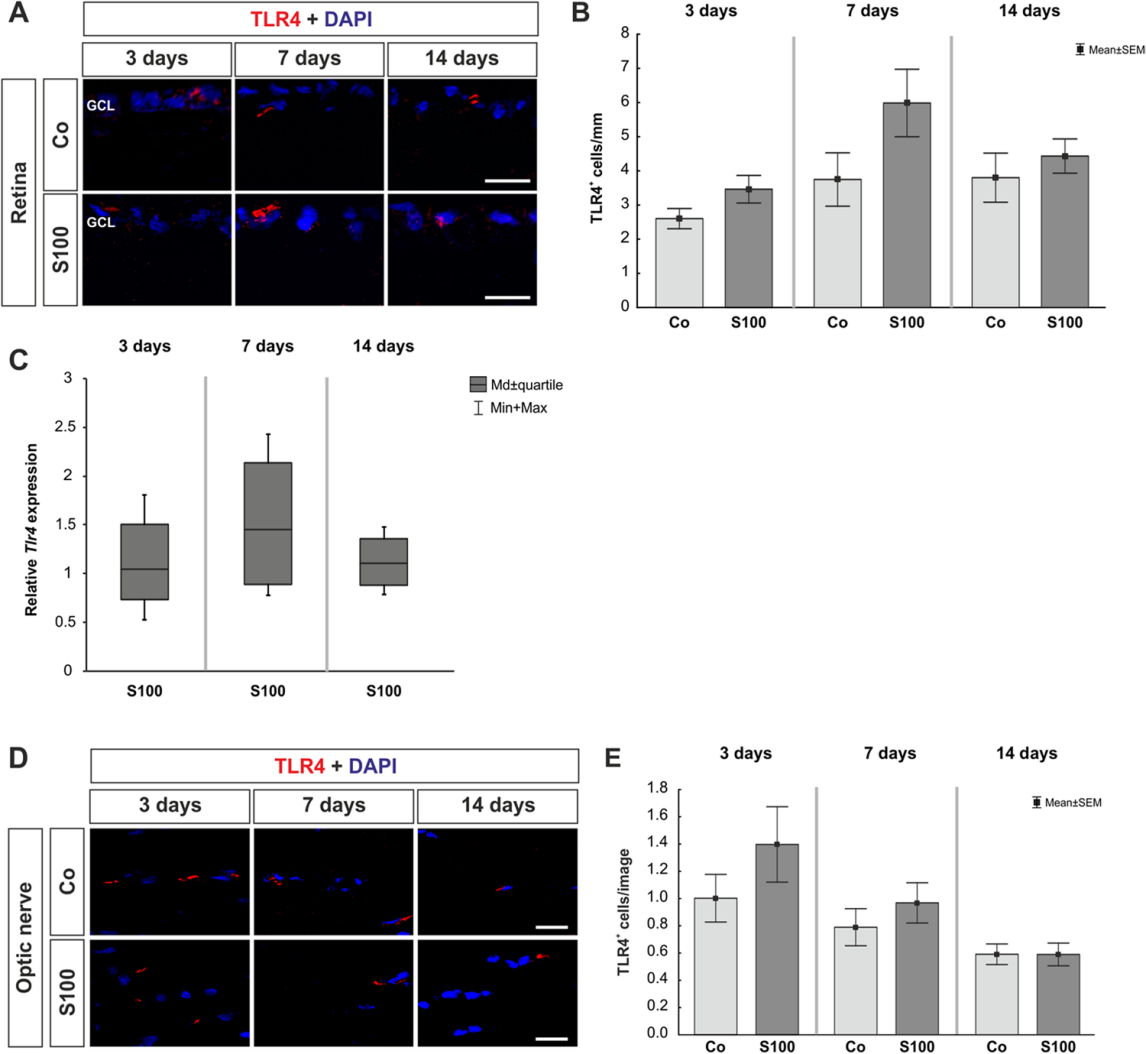
No TLR4 alterations in retina and optic nerve. **(A)** Retinal cross-sections were labeled with an anti-TLR4 antibody (red) and DAPI (blue) at 3, 7, and 14 days. **(B)** No alterations in the number of TLR4^+^ cells were observed in the S100 retinas at all points in time (p > 0.05). **(C)** Also, no altered *Tlr4* mRNA expression could be noted at all points in time via qRT-PCR (p > 0.05). **(D)** To determine TLR4 expression in optic nerves, sections were stained with anti-TLR4 (red) and DAPI (cell nuclei, blue). **(E)** After 3 days, no changes were seen regarding the TLR4 expression in the S100 animals (p > 0.05). Also, no alterations were detected later on (p > 0.05). Abbreviation: GCL = ganglion cell layer. Values are mean ± SEM for immunohistology and median ± quartile + maximum/minimum for qRT-PCR. Scale bars: 20 µm.

The analysis of TLR4^+^ cells in the retinas showed no changes in the S100 group after 3, 7, and 14 days (p > 0.05, Fig. 5A,B). Also, the expression of *Tlr4* mRNA remained unaltered in the S100 retinas at all points in time (p > 0.05, Fig. 5C, Table 1). The quantification of TLR4^+^ cells in the optic nerves also revealed no differences between both groups at all points in time (p > 0.05, Fig. 5D,E).

### Enhanced levels of IL-1β in aqueous humor

**Figure 6:**
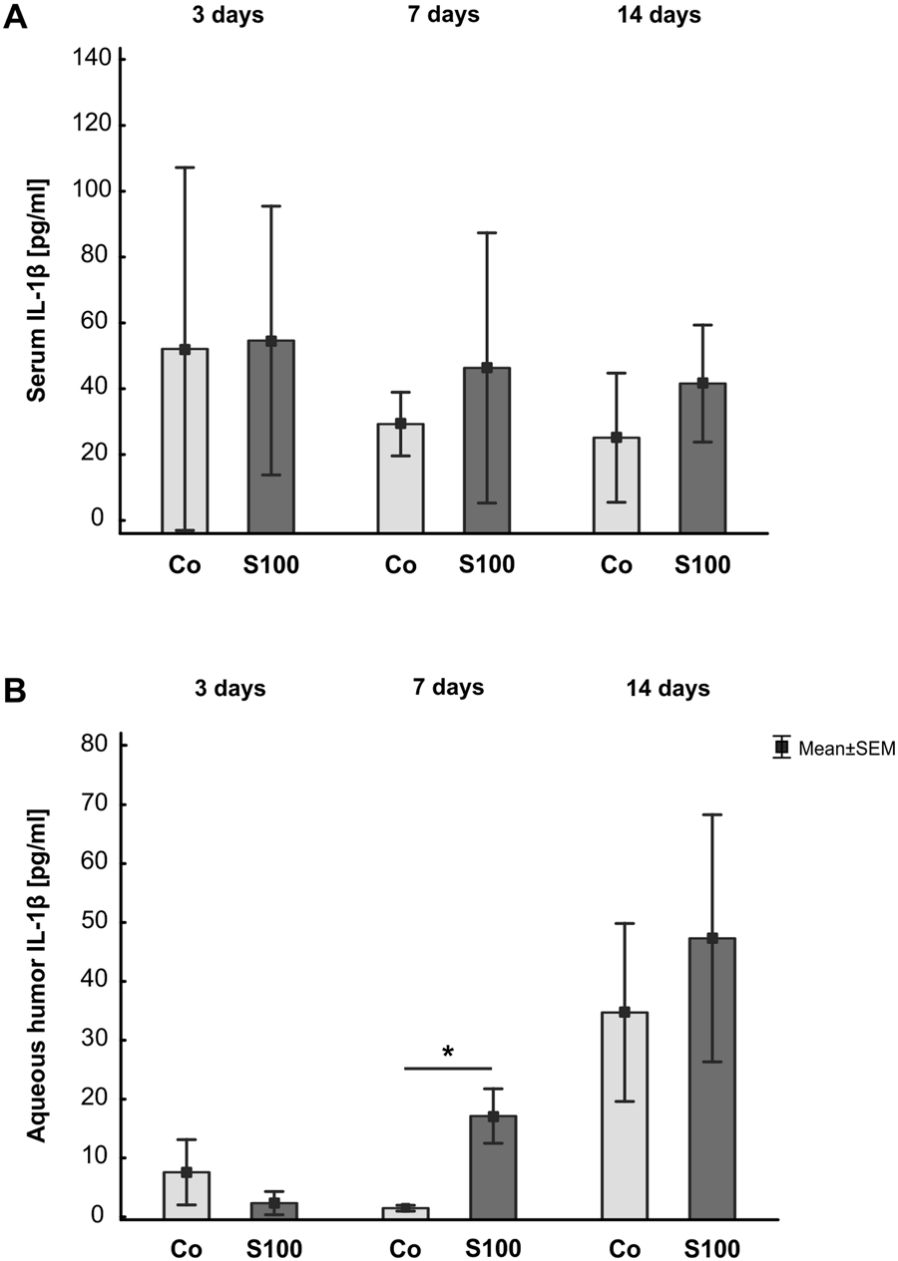
Increased IL-1β levels in aqueous humor. **(A)** The concentration level of IL-1β was measured in rat serum at 3, 7, and 14 days after immunization. The levels of IL-1β were comparable in both groups at all points in time. **(B)** IL-1β was also analyzed in aqueous humor 3, 7, and 14 days after immunization. While no changes were noted at 3 days (p > 0.05), a significant increase of IL-1β could be observed in the S100 group after 7 days (p = 0.03). At 14 days, the IL-1β concentration level was comparable in both groups. Values are mean ± SEM.

ELISA analysis of IL-1β in sera of S100B animals revealed comparable levels as in control animals at all points in time (p > 0.05, Fig. 6A). Also, the concentration levels of IL-1β in the aqueous humor were comparable in both groups at 3 and 14 days (p > 0.05, Fig. 6B). Nevertheless, 7 days after immunization, a significantly higher concentration of IL-1β was measured in the aqueous humor of S100B animals (p = 0.03).

## Discussion

### Complement activation in glaucoma pathogenesis

Several studies indicate an involvement of the immune system in the pathogenesis of glaucoma. Besides findings of autoantibody patterns and depositions, also an activation of microglia was noted in both human glaucoma^4,5,29^ and in experimental animal studies^8,11^. Therefore, a contribution of the complement system in glaucoma disease seems likely. In the study presented here, we noted an activation of the complement system via the lectin pathway, before any cell loss or optic nerve degeneration occurred. This is consistent with previous findings from our group, where the complement system was also initiated through the lectin route following immunization with an optic nerve homogenate antigen (ONA)^19^. MBL, an initiator of the lectin pathway, is a C-type lectin belonging to the collectins^30^. An increase of MBL^+^ cells in the retina was observed at 3 days and an enhancement of NFκB at 7 and 14 days. Since collectins are able to trigger NFκB activation, it is possible that in this model NFκB is triggered via MBL, which subsequently might lead to cell death. Interestingly, in human glaucoma as well as in OHT models, the classical pathway was upregulated^17,31,32^. However, proteomic analysis of human glaucoma donor retinas also revealed an upregulation of proteins linked to the lectin pathway^15^. Additionally, we found a slight increase of the alternative pathway via qRT-PCR analyses. An inappropriate alternative pathway response is known to occur in eye diseases like age-related macular degeneration (AMD). Here, factor B related genes are upregulated, while factor H genes are downregulated^33^. Factor H is an inhibitor of the alternative pathway due to cleavage of C3 leading to its inactivation ^34^. In animal models of AMD, an involvement of the lectin pathway is suggested^12,35,36^. These studies revealed that the alternative pathway is not only sufficient for injury in AMD, its initial activation is also triggered via the alternative and the lectin pathway. Due to the fact that both pathways are activated in our model, an interaction of those could be assumed. However, investigations of the alternative route in glaucoma are rare and need to be extended.

### Enhanced NFκB signaling without TLR4 contribution

The transcription factor NFκB is constitutively expressed in the CNS and is activated in various neurological diseases, such as Morbus Alzheimer, Morbus Parkinson, or Huntington’s disease^37–40^. It is known that components of NFκB are TLR induced genes that undergo positive feedback regulation by NFκB^41^. In human glaucomatous retinas, an upregulation of TLRs in microglia and astrocytes was reported^26^. It could also be observed that TLR4 signaling and NFκB activation accompanied by complement upregulation is a key mechanism of neuroinflammation^42^. An increase of NFκB was observed in the trabecular meshwork of glaucoma patients^43,44^. In microglia, NFκB controls migration to the site of injury due to expression of β-integrin CD11a. In our study, we noted an increase of NFκB in the retinas 7 and 14 days after immunization. This is followed by an activation of microglia at 14 days, which was previously described^8^. Furthermore, enhanced levels of the pro-inflammatory cytokine IL-1β were observed in S100 aqueous humor at 7 days. Yoneda *et al*. noted that IL-1β plays an important role in mediating ischemic and excitotoxic damage in glaucomatous retina^45^. Several studies indicate that IL-1β is secreted by microglia in photo-oxidative damage^46–48^, neovascular AMD^49^, retinitis pigmentosa^50^, and retinal detachment^51^. But not only microglia/macrophages, also NFκB was found to play multiple roles in the induction of IL-1β transcription^52^.

Regarding TLR4, we could not note any alterations in retinas and optic nerves in the EAG model throughout the study. Possibly, an upregulation of TLR4 could be observed at subsequent points in time, since a loss of RGCs is not measurable before day 28 in this model^7,8,19^.

Nonetheless, these results strengthen the hypothesis that NFκB plays a crucial role in glaucoma pathogenesis.

## Conclusion

We conclude that the early activation via the lectin complement pathway triggers NFκB signaling with subsequent microglia response in this EAG model. The combination of these mechanisms seems to initiate the IOP-independent RGC death and optic nerve degeneration. Therefore, an inhibition of complement components might lead to protection of RGCs and optic nerve fibers. However, further studies are required to gain more knowledge regarding the interaction of NFκB/complement signaling components and their contribution to glaucoma like RGC death.

## Methods

### Animals

All procedures concerning animals adhered to the ARVO statement for the use of animals in ophthalmic and vision research. All experiments involving animals were approved by the animal care committee of North Rhine-Westphalia, Germany, and were performed in accordance with relevant guidelines and regulations.

Male Lewis rats (Charles River, Sulzfeld, Germany), 6 weeks of age, were used for the experiments and kept under environmentally controlled conditions with free access to chow and water. Detailed observations and health checks, including eye exams, were performed regularly, as described previously^53^.

### Immunizations

Rats received 1 mg/ml S100B (Sigma Aldrich, Munich, Germany)^7,8^. The antigen was mixed with incomplete Freund’s adjuvant (200 µl) plus 3 µg pertussis toxin (both Sigma Aldrich). The animals of the control group (Co) were injected with 0.9% NaCl in Freund’s adjuvant and pertussis toxin.

For tissue dissection, animals were sacrificed with an overdose CO_2_ at 3, 7, and 14 days after immunization.

### Retinal ganglion cell counts via whole mounts

14 days after immunization, eyes were fixed in 4% paraformaldehyde (PFA) for one hour and then prepared as whole mounts (n = 6/group)^19^. The following steps were performed at 20 °C on a thermo shaker (70 rpm). First, the whole mounts were blocked with 10% donkey serum and 0.5% Triton-X in PBS for 90 min. Then, they were incubated with the RGC marker Brn-3a^54^ (1:300; Santa Cruz, CA, USA) overnight, followed by a 2 hour incubation of donkey anti-goat Alexa Flour 488 (1:1000; Dianova, Hamburg, Germany). From each of the four whole mount arms, three photos were captured (central, middle, and peripheral) with an Axiocam HRc CCD camera on an Axio Imager M1 fluorescence microscope (Zeiss, Jena, Germany). Cells were counted using ImageJ software (V 1.43 u; NIH, Bethesda, MD, USA).

### Retina and optic nerve histology

Retinas and optic nerves were fixed in 4% PFA for 1 (retina) or 2 hours (optic nerves), dehydrated in sucrose, and embedded in Tissue Tek (Thermo Fisher, Waltham, CA, USA). Cross-sections of the retina (10 µm) and longitudinal optic nerve sections (4 µm) were cut with a Cryostat (Thermo Fisher) and mounted on Superfrost slides (Thermo Fisher).

### Immunohistology of retinal and optic nerve sections

To identify the different cell types in the retina and the optic nerve, specific immunofluorescence antibodies were applied (retina: n = 5–6/group, optic nerve: n = 6–8/group, 6 sections/staining; Table 2)^19^. Briefly, retina and optic nerve sections were blocked with a solution containing 10–20% donkey and/or goat serum and 0.1% Triton-X in PBS. Primary antibodies were incubated at room temperature overnight. Incubation using corresponding secondary antibodies was performed for 1 h on the next day. Nuclear staining with 4′, 6 diamidino-2-phenylindole (DAPI) was included to facilitate the orientation on the slides. Negative controls were performed by using secondary antibodies only.

**Table 2:** Primary and secondary antibodies used for immunohistochemistry.

Primary antibodies				Secondary antibodies			
Antibody	Company	Tissue	Dilution	Antibody	Company	Tissue	Dilution
Anti-Brn-3a	SySy	Retina	1:1000	Donkey anti-rabbit Alexa Fluor 488	Invitrogen	Retina	1:500
Anti-Brn-3a	Santa-Cruz	Retina	1:100	Goat anti-mouse Alexa Fluor 488	Invitrogen	Retina	1:500
Anti-C3	Cedarlane	Retina	1:500	Goat anti-rabbit IgG Cy3	Linaris	Retina	1:500
		Optic nerve	1:500			Optic nerve	1:500
Anti-C5b-9 (MAC)	Biozol	Retina	1:100	Donkey anti-mouse DyLight 488	Dianova	Retina	1:250
		Optic nerve	1:100	Goat anti-mouse Alexa Fluor 488	Invitrogen	Optic nerve	1:500
Anti-cleaved caspase 3	Sigma-Aldrich	Retina	1:300	Donkey anti-rabbit Alexa Fluor 555	Invitrogen	Retina	1:500
Anti-ED1	Millipore	Retina	1:250	Donkey anti-mouse Alexa Fluor 488	Invitrogen	Retina	1:500
Anti-factor B	TECOmedical	Retina	1:1000	Donkey anti-goat Cy3	Abcam	Retina	1:600
Anti-MBL	Biozol	Retina	1:100	Donkey anti-rabbit Alexa Fluor 555	Invitrogen	Retina	1:500
				Goat anti-rabbit Alexa Fluor 488		Retina	1:600
		Optic nerve	1:100	Goat anti-rabbit Alexa Fluor 488		Optic nerve	1:600
Anti-NeuN	Millipore	Retina	1:500	Donkey anti-chicken Alexa Fluor 488	Jackson ImmunoResearch	Retina	1:500
Anti-NFκB p50	Santa-Cruz	Retina	1:500	Goat anti-mouse Alexa Fluor 488	Invitrogen	Retina	1:600
				Donkey anti-mouse Alexa Fluor 555			1:500
Anti-S100B	Novus Biological	Optic nerve	1:100	Donkey anti-rabbit Alexa Fluor 555	Invitrogen	Optic nerve	1:500
Anti-SMI-32	Biolegend	Optic nerve	1:2000	Goat anti-mouse Alexa Fluor 488	Invitrogen	Optic nerve	1:400
Anti-TLR4	Abcam	Retina	1:400	Donkey anti-rabbit Alexa Fluor 555	Invitrogen	Retina	1:500

### Histological examination of retinas and optic nerves

All photographs were taken with a fluorescence microscope (Axio Imager M1). In the retina, two photos of the peripheral and two of the central part of each section were captured for each point in time. In the optic nerve, three photos were captured (proximal, middle, and distal). The images were transferred to Corel Paint Shop Pro (V13, Corel Corporation, CA, USA) and equal excerpts were cut out. RGCs, cleaved caspase 3^+^ RGCs, complement factors, NFκB^+^, and TLR4^+^ cells were counted using ImageJ software. The neurofilament marker SMI-32 was evaluated via a scoring system ranging from 0 (intact structure) to 2 (loss of structural integrity, fissures, many retraction bulbs)^8,55,56^. Measurement and analysis of S100B area was performed using ImageJ software as described previously^53,57,58^. Briefly, images were transformed into grayscale. To minimize interference with background labeling, a rolling ball radius of 50 pixels was subtracted. Then, for each picture, a suitable lower threshold was set. The ideal threshold was obtained when the grayscale picture and the original one corresponded. Afterwards, the mean value of the lower threshold was calculated, and this number was used for final analysis. The percentage of the labeled area was measured between these defined thresholds: lower threshold: 11.61; upper threshold: 247.44.

### RNA preparation and cDNA synthesis

For RNA preparation, retinas from every point in time were isolated, transferred into lysis buffer containing 2-mercaptoethanol (Sigma-Aldrich), and snap frozen in liquid nitrogen (n = 3–6/group). Total RNA was extracted with the Gene Elute Mammalian Total RNA Miniprep Kit according to the manufacturer’s instructions (Sigma-Aldrich) and digested with RNase-free DNase I (Sigma-Aldrich). Quality and quantity of RNA were assessed by measuring the ratio of absorbance values at 260 and 280 nm (BioSpectrometer®, Eppendorf, Hamburg, Germany). Total RNA (1 µg) was used for reverse transcription with a cDNA synthesis kit (Thermo Fisher Scientific, Waltham, MA, USA).

### Quantitative real-time PCR

The designed oligonucleotides are shown in Table 3. Real-time-PCR (Roche Applied Science, Mannheim, Germany) analyses were performed using SYBR Green I and the Light Cycler® 96 (Roche Applied Science)^19,59,60^. Oligonucleotide concentration was optimized to a final concentration of 200 nM and combined with 200 ng retinal cDNA per well. We set up two reactions per RNA sample (duplicates) with a final volume of 20 µl per single reaction^61–63^. Each qRT-PCR was performed in duplicates from each retina and for each point in time and repeated twice (n = 3–6/group). The average threshold cycle (Ct) values of the two independent experiments were used to calculate the ratios for the target genes^64^. In order to obtain amplification efficiencies of different primer sets, we generated standard curves by a twofold dilution series with template amounts ranging from 5 to 125 ng cDNA per well. The Ct values of two reference genes (*β-actin* and *cyclophilin*) were taken into account.

**Table 3:** Oligonucleotide sequences.

Gene	Forward (F) and reverse (R) oligonucleotides	GenBank acc. no.	Amplicon size
*Bax-*F	gctggacactggacttcctc	NM_017059.2	127 bp
*Bax-*R	actccagccacaaagatggt		
*Bcl-2-*F	gtacctgaaccggcatctg	NM_016993.1	76 bp
*Bcl-2-*R	ggggccatatagttccacaa		
*C3*-F	tcgaaatccctcccaagtc	NM_016994.2	60 bp
*C3*-R	cgatcttcaaggggacaatg		
*C5*-F	tctcaggccaaagagagacc	XM001079130.4	73 bp
*C5*-R	acggtgtttgtatttagcagctt		
*Cyclophilin*-F	tgctggaccaaacacaaatg	M19553.1	88 bp
*Cyclophilin*-R	cttcccaaagaccacatgct		
*Factor b*-F	aagaaggcagaatgcagagc	NM_212466.3	66 bp
*Factor b*-R	ccagaactccccattttcaa		
*Mbl*-F	aggaccaaaaggccaaaaag	NM_012599.2	68 bp
*Mbl*-R	ccatatttgccagcttcacc		
*Myd88*-F	atgaactgaaggaccgcatc	XM_006244087.3	127 bp
*Myd88*-R	cccagttcctttgtctgtgg		
*NEMO*-F	tctgaagaaatgccaacagc	AY392762.1	61 bp
*NEMO*-R	tgtcacctgagctttcacaga		
*Nfκb*-F	ctggcagctcttctcaaagc	NM_001276711.1	70 bp
*Nfκb*-R	ccaggtcatagagaggctcaa		
*Pou4f1-*F	ctccggaccttgagcttct	XM_008770931.2	60 bp
*Pou4f1-*R	tagaagggagagttaaacacagaaca		
*Tlr4*-F	ccttgagaaagtggagaagtcc	NM_019178.1	61 bp
*Tlr4*-R	gctaagaaggcgatacaattcg		
*β-actin*-F	cccgcgagtacaaccttct	NM_031144.3	72 bp
*β-actin*-R	cgtcatccatggcgaact		

The listed oligonucleotide pairs were used in quantitative real-time PCR experiments, while *β-actin* and *cyclophilin* served as housekeeping genes. The predicted amplicon sizes are given. Abbreviations: F = forward, R = reverse, acc. no. = accession number, bp = base pair.

### Measurement of IL-1β in serum and aqueous humor

Levels of the pro-inflammatory cytokine IL-1β in serum (n = 7/group) and aqueous humor (n = 4–6/group) were measured using a commercially available enzyme immunoassay kit 3, 7, and 14 days after immunization (ELISA; R&D systems, MN, USA). All samples were used undiluted. Each assay was performed according to the manufacturer’s instruction. All measurements were performed on a microplate reader (AESKU Reader with Gen5 ELISA software, AESKU.DIAGNOSTICS, Wendelsheim, Germany)^65^.

### Statistics

Immunohistological and ELISA data are presented as mean ± SEM. The S100 group was compared to the Co group via two-tailed Student’s t-test using Statistica Software (Version 13, Dell, Tulsa, OK, USA). Regarding qRT-PCR, data are presented as median ± quartile + minimum/maximum and were assessed using REST© software (Qiagen, Hilden, Germany)^64^. P-values below 0.05 were considered statistically significant. *p < 0.05, **p < 0.01, ***p < 0.001.

## Electronic supplementary material

Supplementary material
