# Supplementary material for: S100B immunization triggers NFκB and complement activation in an autoimmune glaucoma model
Source: Sci Rep. 2018 Jun 29;8:9821. doi: 10.1038/s41598-018-28183-6 (PMC6026137; doi:10.1038/s41598-018-28183-6)
Supplement: Supplementary file 1 — Supplementary material [file 41598_2018_28183_MOESM1_ESM.docx]

**S100B immunization triggers NFκB and complement activation in an autoimmune glaucoma model**

Sabrina Reinehr^1^, Jacqueline Reinhard^2^, Marcel Gandej^1^, Ivo Gottschalk^1^, Gesa Stute^1^, Andreas Faissner^2^, H Burkhard Dick^1^, Stephanie C Joachim^1^

1: Experimental Eye Research Institute, University Eye Hospital, Ruhr-University Bochum, In der Schornau 23-25, 44892 Bochum, Germany

2: Department of Cell Morphology and Molecular Neurobiology, Faculty of Biology and Biotechnology, Ruhr-University Bochum, Universitaetsstrasse 150, 44780 Bochum, Germany

**Corresponding author**

PD Dr. Stephanie C. Joachim

Experimental Eye Research Institute, University Eye Hospital, Ruhr-University Bochum

In der Schornau 23-25, 44892 Bochum, Germany

Phone: +49-234-299-3156, Fax: +49-234-299-3157

E-Mail: stephanie.joachim@rub.de

**Supplement figure 1:** No RGC loss or signs of apoptosis. **A)** At 14 days, retinal whole mounts were stained with an anti-Brn-3a antibody (green). **B)** The total cell density of Brn-3a^+^ cells showed no changes in S100 animals (p>0.05). Also, the evaluation of the different regions (central, middle, peripheral) revealed no alterations. However, a decline of about 10% of RGCs could be noted. **C)** At 3, 7, and 14 days, cross-sections were labeled with an anti-Brn-3a antibody (green) and apoptotic cells were stained with anti-cleaved caspase 3 (red). DAPI (blue) labeled cell nuclei. **D)** The number of Brn-3a^+^ cells remained unaltered in S100 retinas at all points in time (p>0.05). **E)** Furthermore, no changes in the number of cleaved caspase 3^+^ RGCs could be observed (p>0.05). **F)** The qRT-PCR analyses revealed no changes in the *Pou4f1* mRNA expression at 3, 7, and 14 days. **G)** Additionally, the expression level of *Bax/Bcl-2* showed no alterations in the S100 retinas (p>0.05). Abbreviation: GCL=ganglion cell layer. Values are mean±SEM for immunohistology and median±quartile+ maximum/minimum for qRT-PCR. Scale bars: 20 µm.

**Supplement figure 2:** No differences in MAC expression. **A)** Retinas were labeled with anti-MAC (green) at 3, 7, and 14 days. DAPI was added to visualize cell nuclei (blue). **B)** No changes in the number of MAC^+^ cells were noted in the S100 group at all points in time (p>0.05). **C)** The qRT-PCR analysis of *C5* mRNA revealed no alterations at 3, 7, and 14 days (p>0.05). **D)** Sections of the optic nerves were stained with anti-MAC (green) and DAPI (blue) 3, 7, and 14 days after immunization. **E)** At all points in time, no alterations in the number of MAC^+^ depositions could be noted (p>0.05). Abbreviation: GCL=ganglion cell layer. Values are mean±SEM for immunohistology and median±quartile+maximum/minimum for qRT-PCR. Scale bars: 20 µm.

**Supplement figure 3:** Co-stainings of several markers in the retina 14 days after immunization. DAPI (blue) labeled cell nuclei. MAC depositions (red) in the GCL were mostly co-localized with Brn-3a^+^ cells (green). In contrast, not all NFκB^+^ cells (red) were Brn-3a^+^ (green). MBL (red) was mostly localized in near proximity to NeuN^+^ neurons (green). Additionally, the co-labeling of MBL (red) with the active microglia marker ED1 (green) revealed a co-localization (arrow). Abbreviations: GCL=ganglion cell layer, IPL=inner plexiform layer, INL=inner nuclear layer. Scale bars: 20 µm.

**Supplement figure 1:**

**
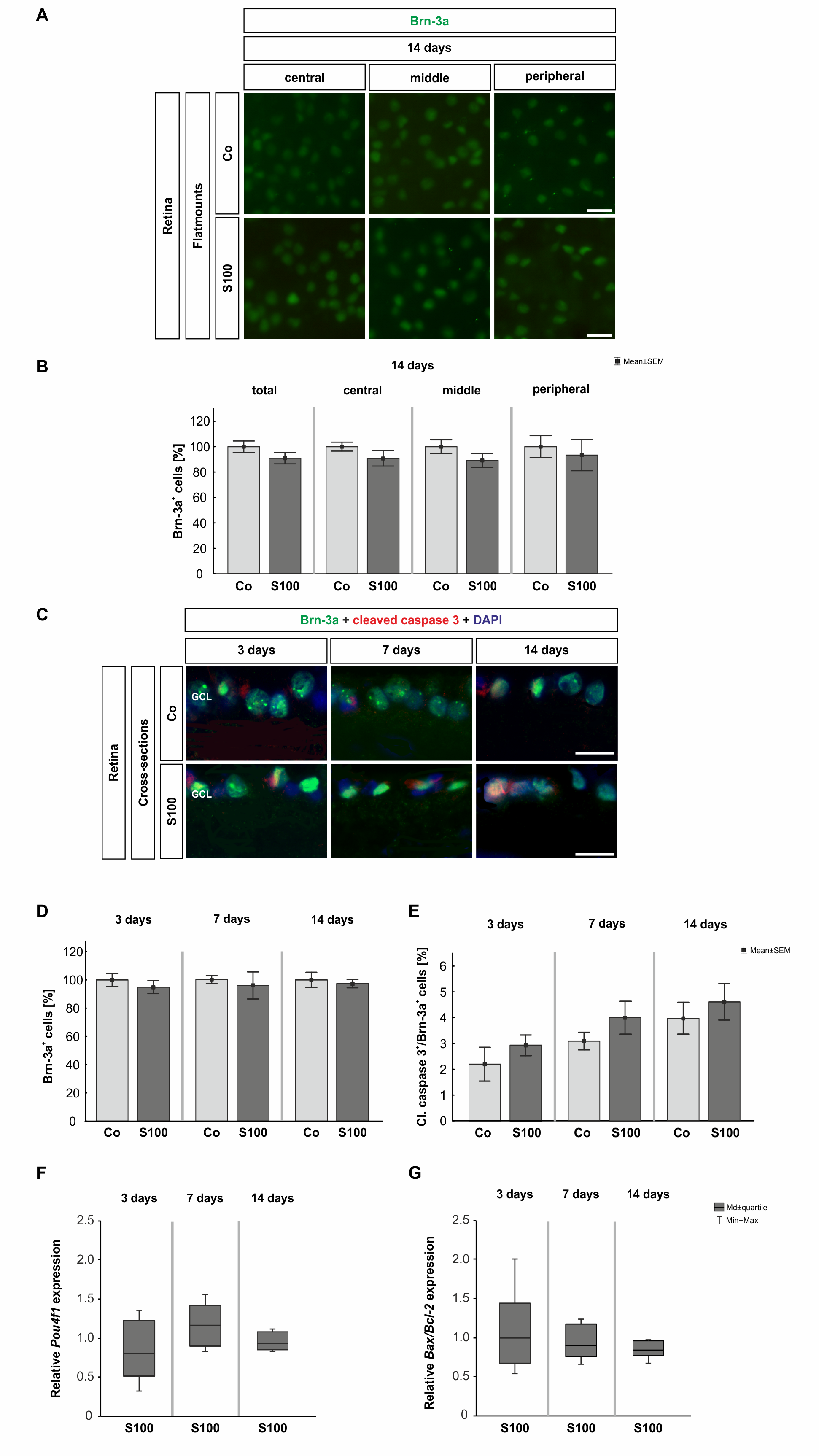
**

**Supplement figure 2:**

**
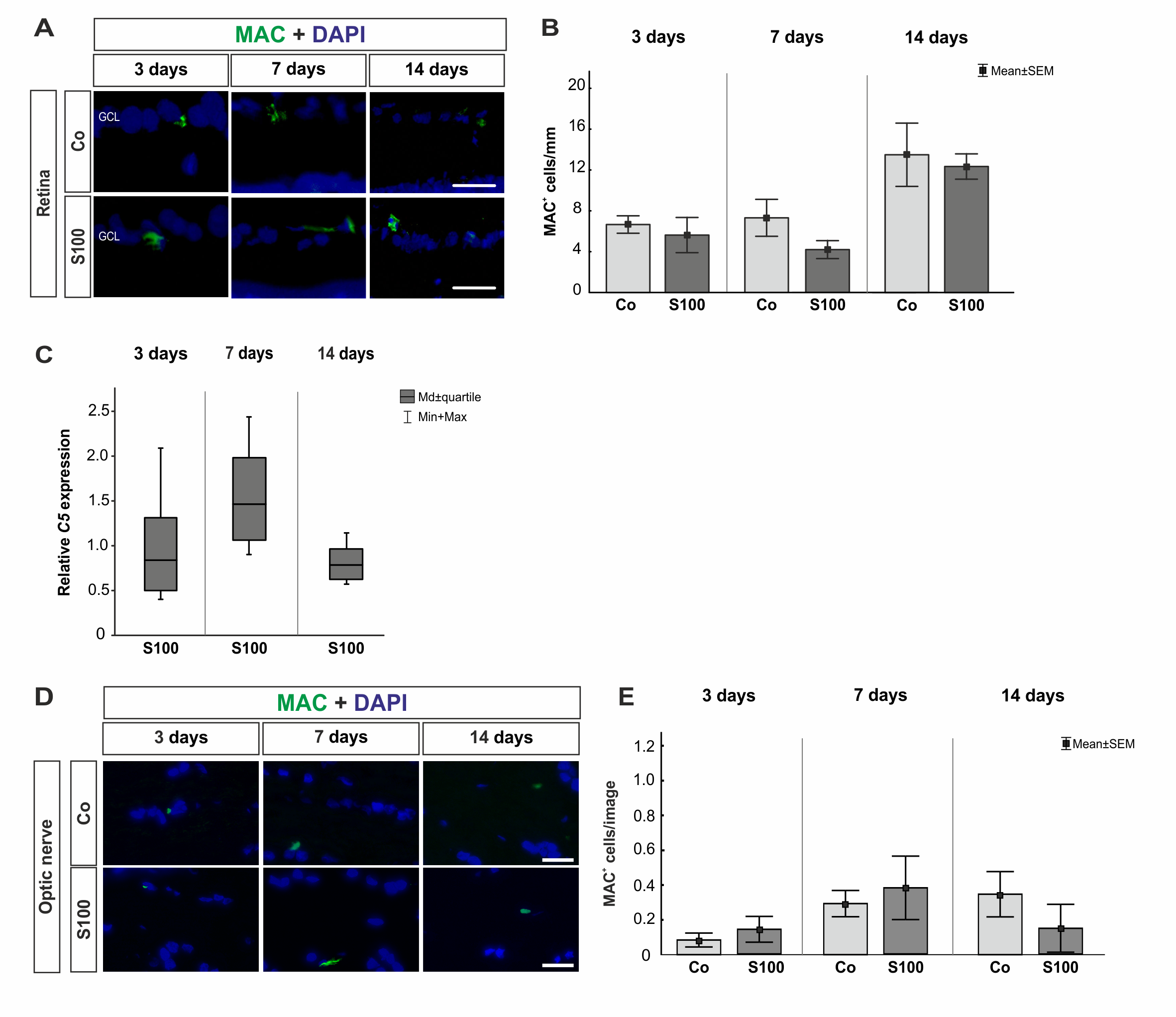
**

**Supplement figure 3:**

**
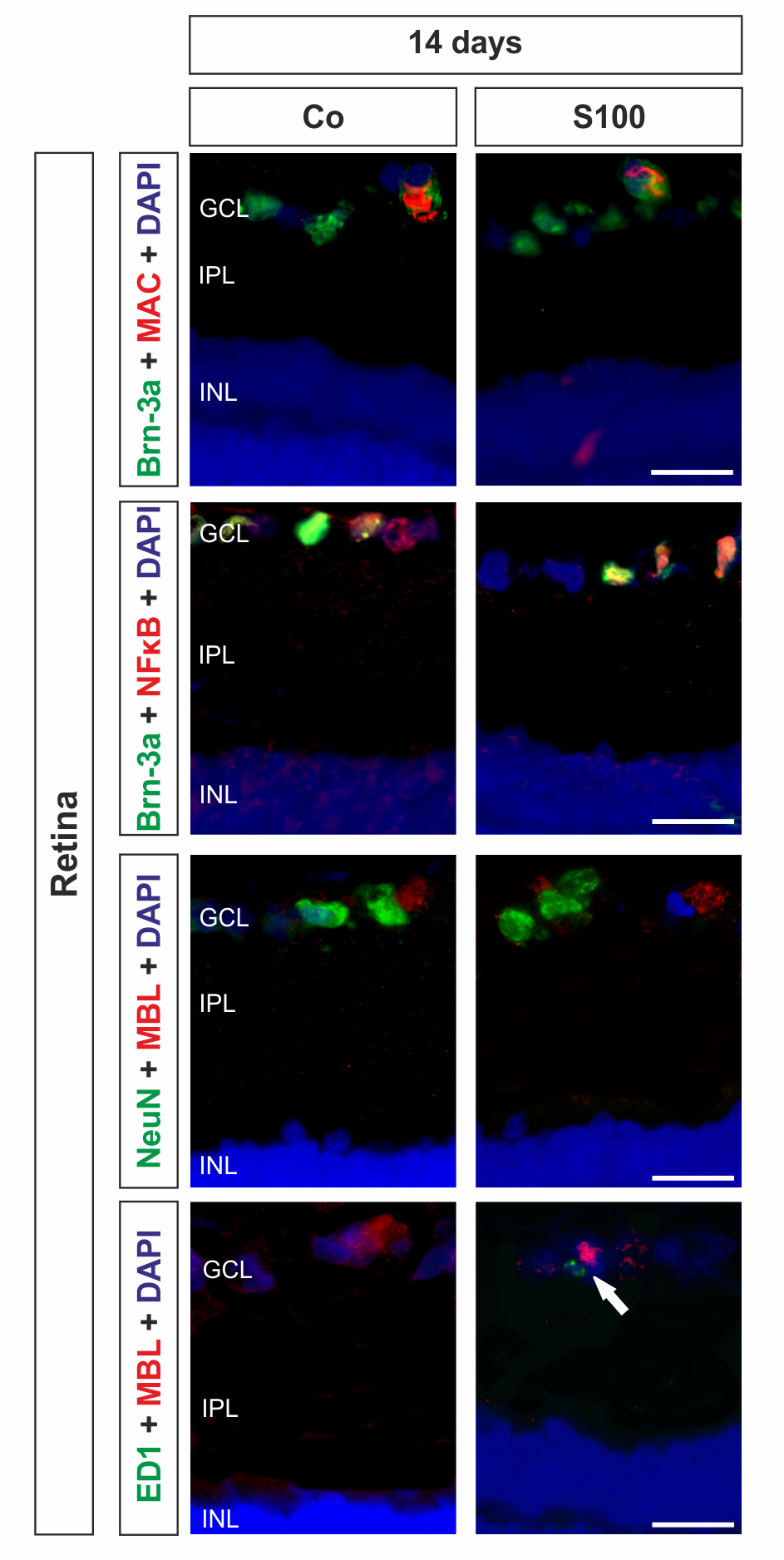
**
